# Logarithmic Sobolev Inequality and Exponential Convergence of a Markovian Semigroup in the Zygmund Space

**DOI:** 10.3390/e20040220

**Published:** 2018-03-23

**Authors:** Ichiro Shigekawa

**Affiliations:** Department of Mathematics, Graduate School of Science, Kyoto University, Kyoto 606-8502, Japan; ichiro@math.kyoto-u.ac.jp; Tel.: +81-75-753-3729

**Keywords:** Dirichlet form, logarithmic Sobolev inequality, entropy, spectrum, Zygmund space, Laguerre operator

## Abstract

We investigate the exponential convergence of a Markovian semigroup in the Zygmund space under the assumption of logarithmic Sobolev inequality. We show that the convergence rate is greater than the logarithmic Sobolev constant. To do this, we use the notion of entropy. We also give an example of a Laguerre operator. We determine the spectrum in the Orlicz space and discuss the relation between the logarithmic Sobolev constant and the spectral gap.

## 1. Introduction

Let (M,B,m) be a measure space with m(M)=1. Suppose we are given a symmetric Dirichlet form E in $L^{2}\left(m\right)$. The associated Markovian semigroup is denoted by $\left\{T_{t}\right\}$ and we assume that $T_{t}1=1$. Here, 1 stands for a constant function of *M*, taking the value 1. For any $f\in L^{1}$, we use the notation

(1) $$\langle f\rangle =\int_{M}f\;dm.$$

We also assume that 1 is the unique invariant function for the semigroup $\left\{T_{t}\right\}$. Then, as t→∞, we have

(2) $$T_{t}f\to \langle f\rangle$$

in $L^{2}\left(m\right)$. The semigroup $\left\{T_{t}\right\}$ is called ergodic when Equation (2) holds. We define the index $\gamma_{2\to 2}$ by

(3) $$\gamma_{2\to 2}=-\bar{\lim}\frac{1}{t}\log{∥T_{t}-m∥}_{2\to 2}$$

which is often called the spectral gap (see e.g., Theorem 4.2.5 of [1]). Here, *m* stands for a linear operator f↦m(f)=〈f〉 and ${∥\;∥}_{2\to 2}$ stands for the operator norm from $L^{2}\left(m\right)$ to $L^{2}\left(m\right)$. In connection to this index, we are interested in another index $\gamma_{Z\to Z}$ defined by

(4) $$\gamma_{Z\to Z}=-\bar{\lim}\frac{1}{t}\log{∥T_{t}-m∥}_{Z\to Z}.$$

Here, *Z* is the Zygmund space (sometimes denoted by LlogL). The space *Z* is defined as follows. Set ϕ(x)=log(1+x) and $\Phi \left(x\right)=\int_{0}^{x}\phi \left(y\right)\;dy$. Then

(5) $$Z=\{f;\int_{M}\Phi (|f|)\;dm<\infty \}.$$

We introduce norms in *Z* later (see Section 2).

On the other hand, the logarithmic Sobolev inequality is a powerful tool in the analysis of Markovian semigroups. The inequality takes the following form:

(6) $$\int_{M}f^{2}\left(x\right)\log(|f\left(x\right)|/{∥f∥}_{2})\;dm\leq \frac{1}{\gamma_{\mathrm{LS}}}E\left(f,f\right).$$

Here, ${∥\;∥}_{2}$ stands for the $L^{2}$-norm and the constant $\gamma_{\mathrm{LS}}$ is chosen to be maximal and is called the logarithmic Sobolev constant. The form of the inequality reminds us the notion of entropy:

(7) Ent(f)=E[flog(f/〈f〉)].

An important application of the logarithmic Sobolev inequality is the following estimate of the entropy (see e.g., Chapter 6.1 of [2]):

(8) $$\mathrm{Ent}\left(T_{t}f\right)\leq e^{-2\gamma_{\mathrm{LS}}\;t}\mathrm{Ent}f.$$

We are interested in the relation between $\gamma_{Z\to Z}$ and $\gamma_{\mathrm{LS}}$. In fact, we show the inequality $\gamma_{Z\to Z}\geq \gamma_{\mathrm{LS}}$. This kind of estimate of $\gamma_{Z\to Z}$ is given in [3], but in this paper we give a direct connection to the constant $\gamma_{\mathrm{LS}}$.

The organization of this paper is as follows. In Section 2, we give several kinds of norms in the Zygmund space *Z*. Using these notions, we show relations between the entropy and the norm in the space *Z* and give a proof of the main result. As an example, we discuss the Laguerre operator in Section 3. We give a precise expression of the resolvent kernel. In Section 4, we introduce Orlicz spaces ($L\log^{\beta }L$). We also discuss how to show the boundedness of operators in Orlicz spaces. Using these, we investigate the spectrum of the Laguerre operator in Orlicz spaces in Section 5. We can completely determine the spectrum and can see the relation between the spectral gap and the logarithmic Sobolev constant.

## 2. Entropy and the Zygmund Space

### 2.1. The Zygmund Space

We start with the Zygmund space. Let (M,B,m) be a measure space, and we assume that m(M)=1, i.e., *m* is a probability measure. All functions in the paper are assumed to be B-measurable. We denote the integration with respect to *m* by 〈f〉. Of course, we assume the integrability of a function *f*. We also use the notation E[f] for 〈f〉.

The Zygmund space is the set of all measurable functions *f* with E[|f|log|f|]<∞. We denote it by *Z* or LlogL. We can define a norm in this space. To do this, we introduce a function ϕ on [0,∞) defined by

(9) ϕ(x)=log(1+x)

and further, we define

(10) $$\Phi \left(x\right)=\int_{0}^{x}\phi \left(y\right)\;dy=\left(1+x\right)\log\left(1+x\right)-x.$$

Φ is a convex function. Now, define $N_{\Phi }$ by

(11) $$N_{\Phi }\left(f\right)=\inf\{\lambda ;E[\Phi (|f|/\lambda )]\leq 1\}.$$

This norm is sometimes called the Luxemburg norm (see e.g., [4]). The norm of the constant function 1 can be computed as

$$\begin{array}{ll} N_{\Phi }\left(1\right) & =\inf\{\lambda ;E[\Phi (1/\lambda )]\leq 1\}\\ & =\inf\{\lambda ;1/\lambda \leq \Phi^{-1}\left(1\right)\}\\ & =\inf\left\{\lambda ;1/\lambda \leq e-1\right\}\;(∵\Phi^{-1}\left(1\right)=e-1)\\ & =\frac{1}{e-1}. \end{array}$$

*Z* becomes a Banach with the norm $N_{\Phi }$.

The dual space of *Z* is given as follows. Let ψ be the inverse function of ϕ, i.e.,

$$\begin{array}{c} \psi \left(x\right)=e^{x}-1. \end{array}$$

Using this, define Ψ by

$$\begin{array}{c} \Psi \left(x\right)=\int_{0}^{x}\psi \left(y\right)\;dy=\int_{0}^{x}\left(e^{y}-1\right)\;dy=e^{x}-x-1. \end{array}$$

The dual space of *Z* can be identified with the space of all measurable functions *f* with E[Ψ(ε|f|)]<∞ for some ε>0 (see [4]).

The following inequality is fundamental:

(12) xy≤Φ(x)+Ψ(y).

Using this, we can show that

(13) $${∥f∥}_{1}\leq \left(e-1\right)N_{\Phi }\left(f\right).$$

In fact, if $N_{\Phi }\left(f\right)=1$, we have

$$\begin{array}{c} E\left[|f|y\right]\leq E[\Phi (|f|)]+E\left[\Psi \left(y\right)\right]=1+\Psi \left(y\right)=e^{y}-y. \end{array}$$

Hence,

$$\begin{array}{c} E\left[|f|\right]\leq \frac{e^{y}}{y}-1. \end{array}$$

The right-hand side takes its minimum e−1 when y=1. Hence, we obtain Equation (13). This shows that $Z\subset L^{1}$.

Further, we have

$$\begin{array}{ll} N_{\Phi }\left(f-\langle f\rangle \right) & \leq N_{\Phi }\left(f\right)+N_{\Phi }\left(\langle f\rangle \right)\\ & =N_{\Phi }\left(f\right)+\left|\langle f\rangle \right|N_{\Phi }\left(1\right)\\ & \leq N_{\Phi }\left(f\right)+{∥f∥}_{1}\frac{1}{e-1}\\ & \leq N_{\Phi }\left(f\right)+N_{\Phi }\left(f\right)\;\left(∵\;\mathrm{Equation}\;(13)\right)\\ & =2N_{\Phi }\left(f\right). \end{array}$$

### 2.2. Entropy

Now, we recall the notion of entropy. In this section, all functions are taken from *Z*. For any non-negative function *f*, the entropy Ent(f) is defined by

(14) Ent(f)=E[flog(f/〈f〉)].

We will discuss the relation between $N_{\Phi }\left(f\right)$ and Ent(f). First, we show the following.

**Proposition** **1.** *For any non-negative function f, we have*

(15) 〈f〉E[Φ(|(f−〈f〉)/〈f〉|)]≤Ent(f).

**Proof.**  We note the following inequality

(16) Φ(|x−1|)≤xlogx−x+1.

Using this, we can get

$$\begin{array}{c} E[\Phi (|\left(f/\langle f\rangle \right)-1|)\leq E\left[\left(f/\langle f\rangle \right)\log\left(f/\langle f\rangle \right)-\left(f/\langle f\rangle \right)+1\right]=\frac{1}{\langle f\rangle }\mathrm{Ent}\left(f\right), \end{array}$$

which is the desired result. ☐

If, in addition, we assume 〈f〉≥1, we can get another estimate.

**Proposition** **2.** *If a non-negative function f satisfies* 〈f〉≥1, *then we have*

(17) E[Φ(|f−〈f〉|)]≤〈f〉Ent(f).

**Proof.**  Let us show the inequality

(18) Φ(|x−〈f〉|)≤〈f〉(xlogx−xlog〈f〉−x+〈f〉)

for any x≥0. Set

$$\begin{array}{c} F(x)=\langle f\rangle (x\log x-x\log\langle f\rangle -x+\langle f\rangle )-\Phi (|x-\langle f\rangle |). \end{array}$$

(1) The case x≥〈f〉.

By the definition,

$$\begin{array}{c} F(x)=\langle f\rangle (x\log x-x\log\langle f\rangle -x+\langle f\rangle )-(1+x-\langle f\rangle )\log(1+x-\langle f\rangle )+x-\langle f\rangle . \end{array}$$

Hence, F(〈f〉)=0. By differentiating the function *F*, we have

$$\begin{array}{c} F^{\prime }\left(x\right)=\langle f\rangle \left(\log x-\log\langle f\rangle \right)-\log\left(1+x-\langle f\rangle \right) \end{array}$$

and so we easily see that $F^{\prime }\left(\langle f\rangle \right)=0$. The second-order derivative is given by

$$\begin{array}{ll} F^{\prime \prime }\left(x\right) & =\frac{\langle f\rangle }{x}-\frac{1}{1+x-\langle f\rangle }\\ & =\frac{\left(\langle f\rangle -1)(x-\langle f\rangle \right)}{x(1+x-\langle f\rangle )}\geq 0. \end{array}$$

Thus, we have F(x)≥0 for x≥〈f〉.

(2) The case x≤〈f〉.

In this case, we have

$$\begin{array}{c} F(x)=\langle f\rangle (x\log x-x\log\langle f\rangle -x+\langle f\rangle )-(1+\langle f\rangle -x)\log(1+\langle f\rangle -x)+\langle f\rangle -x. \end{array}$$

So F(〈f〉)=0 is clear. The derivative of *F* is

$$\begin{array}{cc} F^{\prime }\left(x\right) & =\langle f\rangle (\log x-\log\langle f\rangle )+\log(1+\langle f\rangle -x) \end{array}$$

and so we easily see that $F^{\prime }\left(\langle f\rangle \right)=0$. Furthermore, we have

$$\begin{array}{ll} F^{\prime \prime }\left(x\right) & =\frac{\langle f\rangle }{x}-\frac{1}{1+\langle f\rangle -x}\\ & =\frac{\langle f\rangle +{\langle f\rangle }^{2}-\langle f\rangle x-x}{x(1+\langle f\rangle -x)}\\ & =\frac{\left(\langle f\rangle +1)(\langle f\rangle -x\right)}{x(1+x-\langle f\rangle )}\geq 0. \end{array}$$

Thus, we have $F^{\prime }\left(x\right)\geq 0$ and F(x)≥0 for x≤〈f〉.

Using the inequality Equation (18), we have

$$\begin{array}{c} E[\Phi (|f-\langle f\rangle |)]\leq \langle f\rangle E[f\log f-f\log\langle f\rangle -f+\langle f\rangle )]\leq \langle f\rangle \mathrm{Ent}(f) \end{array}$$

which completes the proof. ☐

Now we are ready to show that the $N_{\Phi }$-norm is dominated by the entropy.

**Proposition** **3.** *For any non-negative function f, we have*

(19) $$N_{\Phi }\left(f-\langle f\rangle \right)\leq \max\left\{\sqrt{\langle f\rangle },\sqrt{\mathrm{Ent}(f)}\right\}\sqrt{\mathrm{Ent}(f)}.$$

**Proof.**  We note that since Φ is convex and Φ(0)=0, Φ satisfies that for J≥1

(20) Φ(Jx)≥JΦ(x)

and, for ε≤1

(21) Φ(εx)≤εΦ(x).

The proof of (19) is divided into two cases.

(1) The case $\langle f\rangle \leq N_{\Phi }\left(f-\langle f\rangle \right)$.

Set $N=N_{\Phi }\left(f-\langle f\rangle \right)$. Applying Proposition 1 to the function $\frac{f}{N}$, we have

$$\begin{array}{ll} \mathrm{Ent}(\frac{f}{N}) & \geq \langle f/N\rangle E[\Phi \left(\frac{\left|((f/N)-\langle f/N\rangle )\right|}{\langle f/N\rangle }\right)]\\ & \geq \langle f/N\rangle \frac{1}{\langle f/N\rangle }E[\Phi (|\left(\left(f/N\right)-\langle f/N\rangle \right)|)]\\ & =E[\Phi \left(\frac{\left|f-\langle f\rangle \right|}{N}\right)]\\ & =1. \end{array}$$

We used Equation (20) in the second line. Thus we have, in this case,

$$\begin{array}{c} N_{\Phi }\left(f-\langle f\rangle \right)\leq \mathrm{Ent}\left(f\right). \end{array}$$

(2) The case $\langle f\rangle \geq N_{\Phi }\left(f-\langle f\rangle \right)$.

Set $N=N_{\Phi }\left(f-\langle f\rangle \right)$. Since $\langle \frac{f}{N}\rangle \geq 1$, we can apply Proposition 2 to $\frac{f}{N}$ and obtain

$$\begin{array}{c} E\left[\Phi \left(|\left(f/N\right)-\langle f/N\rangle \right)|\right)]\leq \frac{\langle f\rangle }{N}\mathrm{Ent}\left(\frac{f}{N}\right). \end{array}$$

Now, $N^{2}\leq \langle f\rangle \mathrm{Ent}\left(f\right)$ follows since the left-hand side equals 1. Hence,

$$\begin{array}{c} N_{\Phi }\left(f-\langle f\rangle \right)\leq {\langle f\rangle }^{1/2}\mathrm{Ent}{\left(f\right)}^{1/2}. \end{array}$$

Combining both of them, we have

$$\begin{array}{c} N_{\Phi }\left(f-\langle f\rangle \right)\leq \max\left\{\mathrm{Ent}{\left(f\right)}^{1/2},{\langle f\rangle }^{1/2}\right\}\mathrm{Ent}{\left(f\right)}^{1/2} \end{array}$$

which completes the proof. ☐

In turn, we prove the inequality of the reversed direction.

**Proposition** **4.** *We have the following inequality*:

(22) $$\mathrm{Ent}\left(f\right)\leq \frac{\langle f\rangle }{\log(4/e)}E[\Phi (|\left(f-\langle f\rangle \right)/\langle f\rangle |)].$$

**Proof.**  Note that

(23) $$\begin{array}{cc} nx\log x-x+1 & =\Phi (|x-1|)\;\mathrm{for}\;x\geq 1, \end{array}$$

(24) $$\begin{array}{cc} x\log x-x+1 & \leq C\Phi (|x-1|)\;\mathrm{for}\;x\in [0,1]. \end{array}$$

Here, $C=\frac{1}{\log(4/e)}$. In fact, Equation (23) is clear and so we only show Equation (24). For x∈[0,1], we have

$$\begin{array}{ll} \Phi (|x-1|) & =\Phi (1-x)\\ & =(1+1-x)\log(1+1-x)-(1-x)\\ & =(2-x)\log(2-x)+x-1. \end{array}$$

We set

$$\begin{array}{c} f(x)=C\{(2-x)\log(2-x)+x-1\}-x\log x+x-1. \end{array}$$

Then

$$\begin{array}{cc} f(0) & =C(2\log2-1)-1=0,\\ f(1) & =0. \end{array}$$

It is not hard to show that f(x)≥0 for x∈[0,1]. Hence, we have Equation (24).

Since C>1, we have, for all x≥0,

$$\begin{array}{c} x\log x-x+1\leq C\Phi (|x-1|). \end{array}$$

Substituting $x=\frac{f}{\langle f\rangle }$ in it and integrating both hands, we have

$$\begin{array}{c} E\left[\frac{f}{\langle f\rangle }\log\frac{f}{\langle f\rangle }-\frac{f}{\langle f\rangle }+1\right]\leq CE\left[\Phi \left(\left|\frac{f}{\langle f\rangle }-1\right|\right)\right]. \end{array}$$

Hence,

$$\begin{array}{c} E[f\log(f/\langle f\rangle )]\leq C\langle f\rangle E[\Phi (|(f-\langle f\rangle )/\langle f\rangle |]. \end{array}$$

This completes the proof. ☐

When 〈f〉≤1, we can show another inequality.

**Proposition** **5.** *If we assume* 〈f〉≤1, *then we have*

(25) Ent(f)≤E[Φ(|f−〈f〉|)]+2.

**Proof.**  Since 〈f〉≤1, we easily see

(26) flogf≤(1+|f−〈f〉|)log(1+|f−〈f〉|).

Using this, we have

$$\begin{array}{ll} \Phi (|f-\langle f\rangle |) & =(1+|f-\langle f\rangle |)\log(1+|f-\langle f\rangle |)-f-\langle f\rangle \\ & \geq f\log f-f-\langle f\rangle \\ & \geq f\log f-\langle f\rangle \log\langle f\rangle +\langle f\rangle \log\langle f\rangle -f-\langle f\rangle . \end{array}$$

Integrating both hands, we have

$$\begin{array}{c} E[\Phi (|f-\langle f\rangle |)]\geq \mathrm{Ent}(f)+\langle f\rangle \log\langle f\rangle -2\langle f\rangle . \end{array}$$

Now set g(x)=−xlogx+2x on [0,1]. Then, $g^{\prime }\left(x\right)=-\log x-1+2=1-\log x\geq 0$ for x∈[0,1]. Hence, *g* takes its maximum at x=1. Therefore,

$$\begin{array}{c} \langle f\rangle \log\langle f\rangle -2\langle f\rangle \leq g(1)=2. \end{array}$$

Thus we get the desired result. ☐

We are ready to show that the entropy is dominated by the $N_{\Phi }$-norm.

**Proposition** **6.** *We have the following inequality*:

(27) $$\mathrm{Ent}\left(f\right)\leq 3N_{\Phi }\left(f-\langle f\rangle \right).$$

**Proof.**  Since the function Φ is convex, Φ satisfies following inequality. For J≥1, we have

(28) Φ(Jx)≥JΦ(x),

and, for ε≤1,

(29) Φ(εx)≤εΦ(x).

The proof is divided into two cases.

(1) The case $\frac{\langle f\rangle }{N_{\Phi }\left(f-\langle f\rangle \right)}\geq 1$.

For notational simplicity, we denote $N_{\Phi }\left(f-\langle f\rangle \right)$ by *N*. Using Proposition 4 for $\frac{f}{N}$,

$$\begin{array}{ll} \mathrm{Ent}(\frac{f}{N}) & \leq \frac{\langle f/N\rangle }{\log(4/e)}E\left[\Phi \left(\frac{\left|((f/N)-\langle f/N\rangle )\right|}{\langle f/N\rangle }\right)\right]\\ & \leq \frac{\langle f/N\rangle }{\log(4/e)}\frac{1}{\langle f/N\rangle }E[\Phi (|\left(\left(f/N\right)-\langle f/N\rangle \right)|)]\\ & \leq \frac{1}{\log(4/e)}. \end{array}$$

Here we used Equation (29) in the second line.

(2) The case $\frac{\langle f\rangle }{N_{\Phi }\left(f-\langle f\rangle \right)}\leq 1$.

This time we use Proposition 5 and obtain

$$\begin{array}{ll} \mathrm{Ent}(\frac{f}{N}) & \leq E[\Phi (|((f/N)-\langle f/N\rangle )|)]+2\\ & =1+2=3. \end{array}$$

Since $\frac{1}{\log(4/e)}\leq 3$, we have Equation (27).

Let us recall the logarithmic Sobolev inequality:

(30) $$\int_{M}f^{2}{\left(x\right)\log(f\left(x\right)}^{2}/{∥f∥}_{2}^{2})\;dm\leq \frac{2}{\gamma_{\mathrm{LS}}}E\left(f,f\right),$$

which yields the following entropy estimate:

(31) $$\mathrm{Ent}\left(T_{t}f\right)\leq e^{-2\gamma_{\mathrm{LS}}\;t}\mathrm{Ent}f.$$

Now, we are in a position to prove the following main theorem.

**Theorem** **1.** *We have the following inequality*.

(32) $$\gamma_{\mathrm{LS}}\leq \gamma_{Z\to Z}$$

**Proof.**  We may assume $\gamma_{\mathrm{LS}}>0$. Let *f* be a non-negative function. If $N_{\Phi }\left(f\right)\leq 1$, then we have for sufficiently large *t*

$$\begin{array}{ll} N_{\Phi }\left(T_{t}f-\langle f\rangle \right) & \leq \sqrt{\mathrm{Ent}(T_{t}f)}\left(\sqrt{\mathrm{Ent}(T_{t}f)}\vee \sqrt{\langle f\rangle }\right)\\ & \leq e^{-\gamma_{\mathrm{LS}}t}\sqrt{\mathrm{Ent}(f)}\left(e^{-\gamma_{\mathrm{LS}}t}\sqrt{\mathrm{Ent}(f)}\vee \sqrt{\langle f\rangle }\right)\;\left(∵\mathrm{Equation}\;(31)\right)\\ & \leq e^{-\gamma_{\mathrm{LS}}t}\sqrt{3N_{\Phi }\left(f-\langle f\rangle \right)}\left(e^{-\gamma_{\mathrm{LS}}t}\sqrt{3N_{\Phi }\left(f-\langle f\rangle \right)}\vee \sqrt{\langle f\rangle }\right)\\ & \leq e^{-\gamma_{\mathrm{LS}}t}\sqrt{6N_{\Phi }\left(f\right)}\left(e^{-\gamma_{\mathrm{LS}}t}\sqrt{6N_{\Phi }\left(f\right)}\vee \sqrt{\left(e-1\right)N_{\Phi }\left(f\right)}\right)\\ & \leq e^{-\gamma_{\mathrm{LS}}t}\sqrt{6(e-1)}. \end{array}$$

Next, we take a general *f*. If $N_{\Phi }\left(f\right)\leq 1$, then $N_{\Phi }(f_{\;+}$), *N*_Φ_(*f*${}_{-}$) ≤ *N*_Φ_(|*f*|) = *N*_Φ_(*f*) ≤ 1 and so

$$\begin{array}{ll} N_{\Phi }\left(T_{t}f-\langle f\rangle \right) & =N_{\Phi }(T_{t}f_{+}-\langle f_{+}\rangle -T_{t}f_{-}+\langle f_{-}\rangle )\\ & \leq N_{\Phi }(T_{t}f_{+}-\langle f\rangle )+N_{\Phi }(T_{t}f_{-}-\langle f_{-}\rangle )\\ & \leq e^{-\gamma_{\mathrm{LS}}t}\sqrt{24(e-1)}. \end{array}$$

Therefore, we have

$$\begin{array}{c} ∥T_{t}{-m∥}_{Z\to Z}\leq \sqrt{24(e-1)}e^{-\gamma_{\mathrm{LS}}t}. \end{array}$$

Hence, this completes the proof. ☐

In Theorem 1, we have shown that $\gamma_{Z\to Z}\geq \gamma_{\mathrm{LS}}$. We now connect the Logarithmic Sobolev constant $\gamma_{\mathrm{LS}}$ and the spectral gap. Let us denote the set of spectrum of A in the Zygmund space *Z* by $\sigma (A_{Z})$. Then, the following inequality is known (see e.g., Chapter IV, Proposition 2.2 of Engel-Nagel [5])

$$\begin{array}{c} \sup\left\{ℜ\lambda ;\;\lambda \in \sigma \left(A_{Z}\right)\right\}\leq \lim_{t\to \infty }\frac{1}{t}\log{∥T_{t}∥}_{Z\to Z}. \end{array}$$

If we restrict ourselves to the mean 0 functions, we have

$$\begin{array}{c} \sup\left\{ℜ\lambda ;\;\lambda \in \sigma \left(A_{Z}\right)\backslash \left\{0\right\}\right\}\leq \lim_{t\to \infty }\frac{1}{t}\log{∥T_{t}-m∥}_{Z\to Z}=-\gamma_{Z\to Z}. \end{array}$$

Now we set

(33) $$\zeta_{\mathrm{SG}}=-\sup\left\{ℜ\lambda ;\;\lambda \in \sigma \left(A_{Z}\right)\backslash \left\{0\right\}\right\}$$

and call it the spectral gap in *Z*. Hence, we have the following inequalities:

(34) $$\gamma_{\mathrm{LS}}\leq \gamma_{Z\to Z}\leq \zeta_{\mathrm{SG}}.$$

**Example** **1.** *Let us consider the Ornstein–Uhlenbeck operator* $A=\frac{d^{2}}{dx^{2}}-x\frac{d}{dx}$ on R. The reference measure is $m\left(dx\right)=\frac{1}{\sqrt{2\pi }}e^{-x^{2}/2}dx$ *and the Dirichlet form is given by*

$$\begin{array}{c} E\left(f,g\right)=\int_{R}f^{\prime }\left(x\right)g^{\prime }\left(x\right)m\left(dx\right). \end{array}$$

*In this case, it is known that* $\gamma_{\mathrm{LS}}=1$. *Moreover* f(x)=x *is an eigenfunction for the eigenvalue* −1. *Hence, we have*

$$\begin{array}{c} -1\leq -\zeta_{\mathrm{SG}}\leq -\gamma_{Z\to Z}\leq -\gamma_{\mathrm{LS}}=-1 \end{array}$$

*which shows* $\gamma_{\mathrm{LS}}=\gamma_{Z\to Z}=\zeta_{\mathrm{SG}}$. *In Section 5, we will give an example that* $\zeta_{\mathrm{SG}}>\gamma_{\mathrm{LS}}$ *holds*.

## 3. Spectrum of the Laguerre Operator

In this section, we give an example.

### 3.1. The Laguerre Operator

We consider the following operator:

(35) $$A=x\frac{d}{dx^{2}}+\left(\alpha +1-x\right)\frac{d}{dx}$$

Since eigenfunctions of A are Laguerre polynomials (see e.g., Lebedev [6]), we call the diffusion process generated by A the Laguerre process as in [7]. It is also an interest rate model called the Cox-Ingersoll-Ross process in mathematical finance.

We assume that α>−1. This is necessary to ensure that the invariant measure becomes a probability measure. Actually, the invariant probability measure is given by

(36) $$m\left(dx\right)=\frac{1}{\Gamma (\alpha +1)}x^{\alpha }e^{-x}dx,$$

which is the gamma distribution of the parameters α+1, 1.

There is another characterization of a diffusion process by a speed measure and a scale function. In our case, setting

(37) $$\rho \left(x\right)=\frac{1}{\Gamma (\alpha +1)}x^{\alpha }e^{-x},$$

the speed measure is m=ρ(x)dx and the scale function *s* is determined by $ds=\frac{1}{x\rho (x)}\;dx$. Following Feller, the boundary 0 is classified as a non-exit, an entrance when α≥0 and exit, and an entrance when −1<α<0. We impose the Neumann boundary condition when −1<α<0 to ensure that the associated diffusion process is conservative.

We can give the associated Dirichlet form E as

(38) $$E\left(u,v\right)=\int_{0}^{\infty }\frac{du}{ds}\frac{dv}{ds}\;ds.$$

Here, $\frac{du}{ds}=x\rho \left(x\right)\frac{du}{dx}$. Therefore,

$$\begin{array}{ll} \int_{0}^{\infty }\frac{du}{ds}\frac{dv}{ds}\;ds & =\int_{0}^{\infty }x\rho \left(x\right)\frac{du}{dx}x\rho \left(x\right)\frac{dv}{dx}\frac{1}{x\rho (x)}\;dx\\ & =\int_{0}^{\infty }x\frac{du}{dx}\frac{dv}{dx}\rho \left(x\right)\;dx\\ & =\int_{0}^{\infty }x\frac{du}{dx}\frac{dv}{dx}\;m\left(dx\right). \end{array}$$

This means that

(39) $$E\left(u,v\right)=\int_{0}^{\infty }xu^{\prime }\left(x\right)v^{\prime }\left(x\right)\;m\left(dx\right).$$

It is well-known that the set of the spectrum of A in $L^{2}\left(m\right)$ is $-Z_{\;+}$ and eigenfunctions are Laguerre polynomials. We also give an expression of the resolvent. To do this, we need confluent hypergeometric functions.

### 3.2. Confluent Hypergeometric Functions

We recall confluent hypergeometric functions (see, e.g., Beals-Wong [8] or Lebedev [6]). They are defined by

(40) $${}_{1}F_{1}\left(a;c;x\right)=\sum_{n=0}^{\infty }\frac{{\left(a\right)}_{n}}{{\left(c\right)}_{n}n!}x^{n}.$$

Here, ${\left(a\right)}_{n}$ is the Pochhammer symbol, i.e.,

(41) $${\left(a\right)}_{n}=\frac{\Gamma (a+n)}{\Gamma (a)}=\left\{\begin{array}{cc} a(a+1)\cdots (a+n-1) & n\geq 1\\ 1 & n=0 \end{array}\right.$$

A function defied by Equation (38) converges for all x∈C and is an analytic function. This function satisfies the following differential equation:

(42) $$xu^{\prime \prime }+\left(c-x\right)u^{\prime }=au.$$

This equation is called the Kummer equation (or the confluent hypergeometric equation), and, of course, is closely related to our generator A in Equation (35). Our interest is in the spectrum of A, and so confluent hypergeometric functions are candidates of eigenfunctions. If ${}_{1}F_{1}$ belongs to $L^{2}$, it is an eigenfunction and it is so when a=−n, $n\in Z_{\;+}$. In this case, ${}_{1}F_{1}$(−*n*; *c*; *x*) is nothing but a Laguerre polynomial (up to constant) and is an eigenfunction. For simplicity, we introduce the following notation:

(43) $$\begin{array}{l} M\left(a,1+\alpha ;x\right)={}_{1}F_{1}\left(a;1+\alpha ;x\right). \end{array}$$

By the way, Equation (42) is a second-order differential equation; there is another independent solution, which is given by

(44) $$U\left(a,1+\alpha ;x\right)=\frac{\Gamma (-\alpha )}{\Gamma (a-\alpha )}M\left(a,1+\alpha ;x\right)+\frac{\Gamma (\alpha )}{\Gamma (a)}x^{-\alpha }M\left(a-\alpha ,1-\alpha ;x\right).$$

This function is called a confluent hypergeometric function of the second kind. Their Wronskian is

(45) $$W\left(M\left(a,1+\alpha ;\;\cdot \;\right)U\left(a,1+\alpha ;\;\cdot \;\right)\right)\left(x\right)=-\frac{\Gamma (1+\alpha )}{\Gamma (a)}x^{-\alpha -1}e^{x}.$$

The Laguerre polynomial is written as

(46) $$L_{n}^{\alpha }\left(x\right)=\frac{{\left(\alpha +1\right)}_{n}}{n!}M\left(-n,\alpha +1;x\right).$$

Our parameter α is chosen to be consistent with the parameter of the Laguerre polynomial. The asymptotic behavior of these function is given as follows (see e.g., Lebedev [6]). When x→0,

(47) $$\begin{array}{cc} M(a,1+\alpha ;x) & \to 1, \end{array}$$

(48) $$\begin{array}{cc} U(a,1+\alpha ;x) & ∼\frac{\Gamma (\alpha )}{\Gamma (a)}x^{-\alpha }. \end{array}$$

However, when α=0, $x^{-\alpha }$ should be replaced by logx.

When x→∞,

(49) $$\begin{array}{cc} M(a,1+\alpha ;x) & ∼\frac{\Gamma (1+\alpha )}{\Gamma (a)}e^{x}x^{a-1-\alpha }, \end{array}$$

(50) $$\begin{array}{cc} U(a,1+\alpha ;x) & ∼x^{-a}. \end{array}$$

Here, we assumed *a*, 1+α≠0,−1,−2,⋯.

Now we can give an expression of the resolvent. Recall that we assumed α>0 and a≠0,−1,−2,⋯. The resolvent $G_{a}={\left(a-A\right)}^{-1}$ has the following kernel expression:

(51) $$G_{a}f\left(x\right)=\int_{0}^{\infty }G_{a}\left(x,y\right)f\left(y\right)\;dy$$

where

(52) $$G_{a}\left(x,y\right)=\left\{\begin{array}{cc} -M\left(a,1+\alpha ;y\right)U\left(a,1+\alpha ,x\right)\frac{1}{p(y)W(y)} & y<x,\\ -M\left(a,1+\alpha ;x\right)U\left(a,1+\alpha ,y\right)\frac{1}{p(y)W(y)} & y>x. \end{array}\right.$$

Here, *W* stands for the Wronskian in Equation (45) and p(y)=y. Hence, we have

(53) $$G_{a}\left(x,y\right)=\left\{\begin{array}{cc} \frac{\Gamma (a)}{\Gamma (1+\alpha )}M\left(a,1+\alpha ;y\right)U\left(a,1+\alpha ,x\right)e^{-y}y^{\alpha } & y<x,\\ \frac{\Gamma (a)}{\Gamma (1+\alpha )}M\left(a,1+\alpha ;x\right)U\left(a,1+\alpha ,y\right)e^{-y}y^{\alpha } & y>x. \end{array}\right.$$

$G_{a}$ is a bounded operator in $L^{2}\left(m\right)$ if a≠0,−1,−2,⋯. We will discuss later what happens in the Zygmund space.

### 3.3. The Logarithmic Sobolev Inequality

We show that the logarithmic Sobolev inequality holds for the Laguerre operator A. You can also see the result in Chapter 2.7.3 of [1] from the view point of the curvature dimension condition. Recall that the Dirichlet form associated with A is given by Equation (39).

**Theorem** **2.** *We assume that* $\alpha >-\frac{1}{2}$. *Then, the following logarithmic Sobolev inequality holds for the Dirichlet form* E *in* (36):

(54) $$\int_{0}^{\infty }u^{2}\log(|u|/{∥u∥}_{2})\nu \left(dx\right)\leq 2E\left(u,u\right).$$

**Proof.**  It is enough to check Bakry-Emery’s $\Gamma_{2}$-criterion. It is as follows. From Equation (36), the square field Γ is given by

(55) $$\Gamma \left(f,g\right)=xf^{\prime }\left(x\right)g^{\prime }\left(x\right).$$

The generator is $Au=xu^{\prime \prime }+\left(\alpha -x\right)u^{\prime }$. Hence, the $\Gamma_{2}$ is computed as

$$\begin{array}{ll} 2\Gamma_{2}\left(u,u\right) & =A\Gamma (u,u)-2\Gamma (Au,u)\\ & =A\left(x{u^{\prime }}^{2}\right)-2x{\left(Au\right)}^{\prime }u^{\prime }\\ & =x{\left(x{u^{\prime }}^{2}\right)}^{\prime \prime }+\left(\alpha -x\right){\left(x{u^{\prime }}^{2}\right)}^{\prime }-2x{\left(xu^{\prime \prime }+\left(\alpha -x\right)u^{\prime }\right)}^{\prime }u^{\prime }\\ & =x{\left({u^{\prime }}^{2}+2xu^{\prime }u^{\prime \prime }\right)}^{\prime }+\left(\alpha -x\right)\left({u^{\prime }}^{2}+2xu^{\prime }u^{\prime \prime }\right)-2xu^{\prime }\left(u^{\prime \prime }+xu^{\prime \prime }-u^{\prime }+\left(\alpha -x\right)u^{\prime \prime }\right)\\ & =x\left(2u^{\prime }u^{\prime \prime }+2u^{\prime }u^{\prime \prime }+2x{u^{\prime }}^{2}+2xu^{\prime }u^{\prime \prime }\right)+\left(\alpha -x\right)\left({u^{\prime }}^{2}+2xu^{\prime }u^{\prime \prime }\right)\\ & \;-2x(u^{\prime }u^{\prime \prime }+xu^{\prime }u^{\prime \prime \prime }-u^{\prime 2}+\left(\alpha -x\right)u^{\prime }u^{\prime \prime })\\ & =2xu^{\prime }u^{\prime \prime }+2x^{2}{u^{\prime \prime }}^{2}+\left(\alpha +x\right){u^{\prime }}^{2}\\ & =2{\left(xu^{\prime \prime }+\frac{1}{2}u^{\prime }\right)}^{2}-\frac{1}{2}{u^{\prime }}^{2}+\left(\alpha +x\right){u^{\prime }}^{2}\\ & =2{\left(xu^{\prime \prime }+\frac{1}{2}u^{\prime }\right)}^{2}+\left(\frac{2\alpha -1}{2x}+1\right)x{u^{\prime }}^{2}. \end{array}$$

Thus, we have

$$\begin{array}{cc} \Gamma_{2}\left(u,u\right) & ={\left(xu^{\prime \prime }+\frac{1}{2}u^{\prime }\right)}^{2}+\frac{1}{2}\left(\frac{2\alpha -1}{2x}+1\right)\Gamma \left(u,u\right). \end{array}$$

From this we have $\Gamma_{2}\left(u,u\right)\geq \frac{1}{2}\Gamma \left(u,u\right)$ under the condition $\alpha \geq \frac{1}{2}$. Due to Bakry-Emery’s $\Gamma_{2}$-criterion, this implies that $\gamma_{\mathrm{LS}}\geq \frac{1}{2}$. ☐

Taking $f\left(x\right)=e^{\xi x}$, we can see that $\gamma_{\mathrm{LS}}=\frac{1}{2}$ is the best constant.

**Remark** **1.** *This result was shown in Korzeniowski-Stroock* [7] *when* α=1. *In that paper, it was emphasized that the logarithmic Sobolev constant differs from the spectral gap*.

## 4. Orlicz Space $L\log^{\beta }L$

We start with the definition of the Orlicz space. Take any β>0 and fix it. We introduce a norm in the space of all functions *f* with $E[|f|\log^{\beta }(1+|f|)]<\infty$. Define a function ϕ on [0,∞) by

(56) ϕ(x)=log(1+x).

Then, further define

(57) $$\Phi_{\beta }\left(x\right)=\int_{0}^{x}\phi^{\beta }\left(y\right)\;dy=\int_{0}^{x}\log^{\beta }\left(1+y\right)\;dy.$$

$\Phi_{\beta }$ is a concave function. To get the behavior of $\Phi_{\beta }$ at *∞*, we use the l’Hospital theorem and get

$$\begin{array}{ll} \lim_{x\to \infty }\frac{\left(x\right)}{x\log^{\beta }\left(1+x\right)} & =\lim_{x\to \infty }\frac{\Phi_{\beta }^{\prime }\left(x\right)}{{\left(x\log^{\beta }\left(1+x\right)\right)}^{\prime }}\\ & =\lim_{x\to \infty }\frac{\log^{\beta }\left(1+x\right)}{\log^{\beta }\left(1+x\right)+\beta x\log^{\beta -1}\left(1+x\right)\frac{1}{1+x}}\\ & =\lim_{x\to \infty }\frac{1}{1+\frac{\beta x}{\left(1+x)\log(1+x\right)}}\\ & =1. \end{array}$$

Therefore, when x→∞, we can see

(58) $$\Phi_{\beta }\left(x\right)∼x\log^{\beta }\left(1+x\right).$$

We define the space $L\log^{\beta }L$ by

(59) $$L\log^{\beta }L=\{f;E[\Phi_{\beta }(|f|)]<\infty \}.$$

Then, $L\log^{\beta }L$ becomes a Banach space with the norm $N_{\Phi_{\beta }}$ defined by

(60) $$N_{\Phi_{\beta }}\left(f\right)=\inf\{\lambda ;E[\Phi_{\beta }(|f|/\lambda )]\leq 1\}.$$

For instance, the norm of the constant function 1 is

$$\begin{array}{c} N_{\Phi_{\beta }}\left(1\right)=\inf\left\{\lambda ;E\left[\Phi \left(1/\lambda \right)\right]\leq 1\right\}=\inf\left\{\lambda ;1/\lambda \leq \Phi^{-1}\left(1\right)\right\}=\frac{1}{\Phi^{-1}\left(1\right)}. \end{array}$$

If β=1, then $\Phi^{-1}\left(1\right)=e-1$. In the sequel, the operator norm of linear operators from $L\log^{\beta }L$ into $L\log^{\beta }L$ is defined by using the norm $N_{\Phi_{\beta }}$.

### 4.1. Dual Space

The dual space of $L\log^{\beta }L$ is characterized as follows. Let $\psi_{\beta }$ be the inverse function of $\log^{\beta }\left(1+x\right)$:

(61) $$\psi_{\beta }\left(x\right)=e^{x^{1/\beta }}-1.$$

Further, we define

(62) $$\Psi_{\beta }\left(x\right)=\int_{0}^{x}\psi_{\beta }\left(y\right)\;dy.$$

The Orlicz space associated with $\Psi_{\beta }$ is the dual space of $L\log^{\beta }L$. Let us study the asymptotic behavior of $\Psi_{\beta }$ at x=∞.

**Proposition** **7.** *We have the following*:

(63) $$\Psi_{\beta }\left(x\right)∼\beta e^{x^{1/\beta }}x^{(\beta -1)/\beta }\;as\;x\to \infty .$$

**Proof.**  We use the l’Hôspital theorem.

$$\begin{array}{ll} \lim_{x\to \infty }\frac{\Psi_{\beta }\left(x\right)}{e^{x^{1/\beta }}x^{(\beta -1)/\beta }} & =\lim_{x\to \infty }\frac{\Psi_{\beta }^{\prime }\left(x\right)}{{\left(e^{x^{1/\beta }}x^{(\beta -1)/\beta }\right)}^{\prime }}\\ & =\lim_{x\to \infty }\frac{e^{x^{1/\beta }}-1}{e^{x^{1/\beta }}\left(1/\beta \right)x^{1/\beta -1}x^{(\beta -1)/\beta }+e^{x^{1/\beta }}\frac{\beta -1}{\beta }x^{-1/\beta }}\\ & =\lim_{x\to \infty }\frac{e^{x^{1/\beta }}-1}{\left(e^{x^{1/\beta }}/\beta \right)+e^{x^{1/\beta }}\frac{\beta -1}{\beta }x^{-1/\beta }}\\ & =\lim_{x\to \infty }\frac{1-e^{-x^{1/\beta }}}{\frac{1}{\beta }+\frac{\beta -1}{\beta }x^{-1/\beta }}\\ & =\beta . \end{array}$$

Equation (63) easily follows from this. ☐

The following Hausdorff-Young inequality plays a fundamental role in the later computation.

(64) $$xy\leq \Phi_{\beta }\left(x\right)+\Psi_{\beta }\left(y\right).$$

For example, if $N_{\Phi }\left(f\right)=1$, then we can show that for y>0

$$\begin{array}{c} E[|f|y]\leq E[\Phi_{\beta }\left(|f|)\right]+E\left[\Psi_{\beta }\left(y\right)\right]=1+\Psi_{\beta }\left(y\right). \end{array}$$

Hence,

$$\begin{array}{c} E\left[|f|\right]\leq \frac{1+\Psi_{\beta }\left(y\right)}{y}. \end{array}$$

This shows that $L\log^{\beta }L\subseteq L^{1}$ and there exists a constant $\kappa_{\beta }>0$ so that

$$\begin{array}{c} {∥f∥}_{1}\leq \kappa_{\beta }N_{\Phi_{\beta }}\left(f\right). \end{array}$$

### 4.2. Linear Operators in Orlicz Spaces

Orlicz space $L\log^{\beta }L$ is a Banach space with the norm $N_{\Phi_{\beta }}$. The operator norm can also be defined in terms of this norm. However, since this norm is hard to calculate concretely, we take another way.

We introduce a new norm ${∥\;∥}_{\Phi }$, which is called the Orlicz norm, by

(65) $${∥f∥}_{\Phi_{\beta }}=\sup\{E[g|f|];E\left[\Psi_{\beta }\left(g\right)\right]\leq 1\}.$$

Here, *g* runs over all functions satisfying $E[\Psi_{\beta }\left(g\right)]\leq 1$. Replacing *f* with f/2,

$$\begin{array}{ll} {∥f/2∥}_{\Phi_{\beta }} & =\sup\{E[g|f/2|];E\left[\Psi_{\beta }\left(g\right)\right]\leq 1\}\\ & =\sup\{E[\left(g/2\right)|f|];E\left[\Psi_{\beta }\left(2g/2\right)\right]\leq 1\}\\ & =\sup\{E[g|f|];E\left[\Psi_{\beta }\left(2g\right)\right]\leq 1\}. \end{array}$$

Hence, we can rewrite Equation (65) as follows:

$$\begin{array}{l} {∥f∥}_{\Phi_{\beta }}=2\sup\{E[g|f|];E\left[\Psi_{\beta }\left(2g\right)\right]\leq 1\}. \end{array}$$

We will rewrite the condition $E[\Psi_{\beta }\left(2g\right)]\leq 1$.

**Proposition** **8.** *We have*

$$\begin{array}{c} \sup\{E\left[e^{g^{1/\beta }}\right];\;g\geq 0\;\mathrm{and}\;E[\Psi_{\beta }\left(2g\right)]\leq 1\}<\infty . \end{array}$$

**Proof.**  From Proposition 7, we have

$$\begin{array}{c} \Psi_{\beta }\left(2x\right)∼\beta e^{{\left(2x\right)}^{1/\beta }}{\left(2x\right)}^{(\beta -1)/\beta }. \end{array}$$

We can take large constant $C_{\beta }$ so that

$$\begin{array}{c} e^{x^{1/\beta }}\leq \Psi_{\beta }\left(2x\right)+C_{\beta }. \end{array}$$

Therefore, if $E[\Psi_{\beta }\left(2g\right)]\leq 1$, then we have

$$\begin{array}{c} E\left[e^{g^{1/\beta }}\right]\leq E\left[\Psi_{\beta }\left(2g\right)\right]+C_{\beta }\leq 1+C_{\beta }, \end{array}$$

which is the desired result. ☐

We set

(66) $$K_{\beta }=\sup\left\{E\left[e^{g^{1/\beta }}\right];\;g\geq 0\;\mathrm{and}\;E[\Psi_{\beta }\left(2g\right)]\leq 1\right\}.$$

Then, by Proposition 8, we can see that $E\left[e^{g^{1/\beta }}\right]\leq K_{\beta }$ if $E[\Psi_{\beta }\left(2g\right)]\leq 1$.

It is well-known that two norms, $N_{\Phi_{\beta }}$ and ${∥\;∥}_{\Phi_{\beta }}$, are equivalent (see e.g., p. 61, Chapter III. 3.3, Proposition 4 of Rao-Ren [4]):

(67) $$N_{\Phi_{\beta }}\left(f\right)\leq {∥f∥}_{\Phi_{\beta }}\leq 2N_{\Phi_{\beta }}\left(f\right).$$

From this, we have the following

**Proposition** **9.** *A linear operator T on* $L\log^{\beta }L$ *is bounded if there exist positive constants A and B so that*

(68) $${∥Tf∥}_{\Phi_{\beta }}\leq AE[\Phi_{\beta }\left(|f|)\right]+B.$$

**Proof.**  If we assume Equation (68), then we have

$$\begin{array}{c} ∥Tf/N_{\Phi_{\beta }}{\left(f\right)∥}_{\Phi_{\beta }}\leq AE[\Phi_{\beta }\left(|f\right|/N_{\Phi_{\beta }}\left(f\right))]+B=A+B, \end{array}$$

which implies ${∥Tf∥}_{\Phi_{\beta }}\leq \left(A+B\right)N_{\Phi_{\beta }}\left(f\right)$. The rest is easy from Equation (67).

**Corollary** **1.** *Let* $K_{\beta }$ *be a constant defined by* (65). *Then a linear operator T on* $L\log^{\beta }L$ *is bounded if there exist constants A and B so that for any non-negative function g with* $E\left[e^{g^{1/\beta }}\right]\leq K_{\beta }$ *and any non-negative function* $f\in L\log^{\beta }L$, *we have*

(69) $$E\left[g|Tf|\right]\leq AE[|f|\log_{+}^{\beta }\left|f|\right]+B.$$

**Proof.**  From Equation (58), we have $\Phi \left(x\right)∼x\log^{\beta }\left(x+1\right)$ and hence we can find constants *a* and *b* so that

$$\begin{array}{c} x\log_{+}^{\beta }x\leq a\Phi_{\beta }\left(x\right)+b. \end{array}$$

Then

$$\begin{array}{ll} {∥Tf∥}_{\Phi_{\beta }} & =2\sup\{E[g|Tf|];E\left[\Psi_{\beta }\left(2g\right)\right]\leq 1\}\\ & \leq 2\sup\{E[g|Tf|];E\left[e^{g^{1/\beta }}\leq K_{\beta }\right\}\\ & \leq 2\{AE[|f|\log_{+}^{\beta }|f|]+B\}\;\left(∵\;\mathrm{Equation}\;(69)\right)\\ & \leq 2AE[|f|\log_{+}^{\beta }|f|]+2B\\ & \leq 2AE[a\Phi_{\beta }(|f|)+b]+2B\\ & \leq 2aAE[\Phi_{\beta }\left(|f|)\right]+2bA+2B. \end{array}$$

Now, from Proposition 9, *T* is bounded. ☐

We list up some inequalities which are necessary later. For *x*, y≥0,

$$\begin{array}{c} {\left(x+y\right)}^{p}\leq 2^{p-1}\left(a^{p}+b^{p}\right),\;p\geq 1,\\ {\left(x+y\right)}^{p}\leq a^{p}+b^{p},\;p\leq 1. \end{array}$$

There exists a positive constant $A_{\beta }$ so that

(70) $$xy\leq A_{\beta }\left(x\log_{+}^{\beta }x+e^{y^{1/\beta }}\right).$$

This inequality is a modification of the following Hausdorff-Young inequality:

$$\begin{array}{c} xy\leq x\log x-x+e^{y}. \end{array}$$

## 5. The Spectrum of the Laguerre Operator in $L\log^{\beta }L$

The kernel representation of the resolvent of Laguerre operator was given in Equation (53). It is bounded in $L^{2}$. We will examine whether it is bounded in $L\log^{\beta }L$. Recall that our reference measure is $m=\frac{1}{\Gamma (\alpha +1)}x^{\alpha }e^{-x}\;dx$. We assume α>−1. From now on, we ignore the constant and consider the measure $x^{\alpha }e^{-x}\;dx$.

We take any $f\in L\log^{\beta }L$. We also take a non-negative function *g* satisfying $E\left[e^{g^{1/\beta }}\right]\leq K_{\beta }$. Our aim is to show that there exist constants *A* and *B* so that $E[g|G_{a}f|]\leq AE[|f|\log_{+}^{\beta }\left|f|\right]+B$. The integrability is important, and we do not need the precise constant. Hence, we use the following notation:

$$\begin{array}{c} x≲y. \end{array}$$

This means that there exist constants *k* and *l* so that

$$\begin{array}{c} x\leq ky+l. \end{array}$$

Here, constants *k* and *l* are independent of functions *f* and *g*. This is important but we do not mention this each time.

We starts with an estimate of the defective Gamma function.

**Proposition** **10.** *Take any* k∈R. *If* k<0, *then*

(71) $$\int_{y}^{\infty }x^{k}e^{-x}\;dx\leq y^{k}e^{-y},\;y\geq 0.$$

*If* k≥0, *then there exists a constant* $c_{k}$ *so that*

(72) $$\int_{y}^{\infty }x^{k}e^{-x}\;dx\leq c_{k}{\left(y+1\right)}^{k}e^{-y},\;y\geq 0.$$

These are easily obtained by seeing that $\int_{y}^{\infty }x^{k}e^{-x}\;dx∼y^{k}e^{-y}\;dx$ as y→∞.

**Proposition** **11.** *Assume that* κ>0 *and* λ>−1. *Then, there exists a constant C depending on κ and λ so that*

(73) $$\int_{0}^{y}{\left(-\log x\right)}^{\kappa }x^{\lambda }\;dx\leq C{\left(-\log y+1\right)}^{\kappa }y^{\lambda +1},\;y\leq 1.$$

**Proof.**  This inequality can be reduced to the previous proposition. By the change of variable formula, we have

$$\begin{array}{ll} \int_{0}^{y}{\left(-\log x\right)}^{\kappa }x^{\lambda }\;dx & =\int_{\infty }^{-(\lambda +1)\log y}{\left(\frac{u}{\lambda +1}\right)}^{\kappa }e^{-\frac{u}{\lambda +1}\lambda }e^{-\frac{u}{\lambda +1}}\;\frac{-du}{\lambda +1}\\ & \;\;\left(u=-\left(\lambda +1\right)\log x,\;x=e^{-u/(\lambda +1)},\;dx=-e^{-u/(\lambda +1)}\frac{du}{\lambda +1}\right)\\ & ={\left(\frac{1}{\lambda +1}\right)}^{\kappa +1}\int_{-(\lambda +1)\log y}^{\infty }u^{\kappa }e^{-u}\;du\\ & \leq C{\left(\frac{1}{\lambda +1}\right)}^{\kappa +1}{\left(-\left(\lambda +1\right)\log y+1\right)}^{\kappa }e^{(\lambda +1)\log y}\;\left(∵\mathrm{Equation}\;(72)\right)\\ & \leq \frac{c_{1}}{\lambda +1}{\left(-\log y+\frac{1}{\lambda +1}\right)}^{\kappa }y^{\lambda +1}\\ & \leq \frac{c_{2}}{\lambda +1}{\left(-\log y+1\right)}^{\kappa }y^{\lambda +1}. \end{array}$$

This completes the proof. ☐

We study integrals involving function *g*.

**Proposition** **12.** *For any* k∈R, α>−1 *and* β>0, *there exist constants* $C_{1}$, $C_{2}$ *so that*

(74) $$\int_{y}^{\infty }g\left(x\right)x^{k}x^{\alpha }e^{-x}\;dx\leq C_{1}e^{-y}y^{k}\int_{y}^{\infty }e^{g{\left(x\right)}^{1/\beta }}x^{\alpha }e^{-x}\;dx+C_{2}y^{k+\beta +\alpha }e^{-y},\;\forall y\geq 1.$$

*We have assumed that* $E\left[e^{g^{1/\beta }}\right]\leq K_{\beta }$, *so we have that there exists a constant c depending only on k, β, and α so that*

(75) $$\int_{y}^{\infty }g\left(x\right)x^{k}e^{\alpha }e^{-x}\;dx\leq cy^{k}y^{(\beta +\alpha )\vee 0}e^{-y},\;\forall y\geq 1.$$

**Proof.**  Set $F\left(x\right)=x^{k}e^{-x}$, x≥0. Then

$$\begin{array}{c} F^{\prime }\left(x\right)=kx^{k-1}e^{-x}-e^{-x}x^{k}=\left(k-x\right)x^{k-1}e^{-x} \end{array}$$

*F* takes its maximum at x=k and for x≥k, *F* is decreasing. Hence, if k≤y≤x, then

$$\begin{array}{c} x^{k}e^{-x}\leq y^{k}e^{-y} \end{array}$$

and if 1≤y≤k, then for y≤x, we have

$$\begin{array}{c} x^{k}e^{-x}\leq F\left(k\right)\leq F\left(k\right)F{\left(1\right)}^{-1}F\left(1\right)\leq F\left(k\right)F{\left(1\right)}^{-1}F\left(y\right)\leq F\left(k\right)F{\left(1\right)}^{-1}y^{k}e^{-y}. \end{array}$$

Hence, there exists a constant $c_{k}$ depending on *k* such that for 1≤y≤x,

(76) $$x^{k}e^{-x}\leq c_{k}y^{k}e^{-y}.$$

Therefore

$$\begin{array}{ll} \int_{y}^{\infty }g\left(x\right)x^{k}x^{\alpha }e^{-x}\;dx & =\int_{y}^{\infty }\left(g\left(x\right)e^{x}\right)e^{-x}x^{k}x^{\alpha }e^{-x}\;dx\\ & \leq A_{\beta }\int_{y}^{\infty }\left(e^{g{\left(x\right)}^{1/\beta }}+e^{x}\log_{+}^{\beta }e^{x}\right)e^{-x}x^{k}x^{\alpha }e^{-x}\;dx\;\left(∵\mathrm{Equation}\;(70)\right)\\ & \leq A_{\beta }\int_{y}^{\infty }e^{g{\left(x\right)}^{1/\beta }}\left(x^{k}e^{-x}\right)x^{\alpha }e^{-x}\;dx+A_{\beta }\int_{y}^{\infty }x^{k+\beta }x^{\alpha }e^{-x}\;dx\\ & \leq A_{\beta }c_{k}y^{k}e^{-y}\int_{y}^{\infty }e^{g{\left(x\right)}^{1/\beta }}x^{\alpha }e^{-x}\;dx+A_{\beta }c_{k+\beta +\alpha }y^{k+\beta }y^{\alpha }e^{-y}.\\ & \;\;(∵\;\mathrm{Proposition}\;10\;\mathrm{and}\;\mathrm{Equation}\;(76)) \end{array}$$

This completes the proof. ☐

**Proposition** **13.** *Assume* k+β+α+1>0. *Then, there exists a constant C so that*

(77) $$\int_{1}^{y}g\left(x\right)x^{k}x^{\alpha }\;dx\leq y^{k+\beta +\alpha +1}\left\{C_{1}+C_{2}\int_{1}^{y}e^{g{\left(x\right)}^{1/\beta }}x^{\alpha }e^{-x}\;dx\right\},\;\forall y\geq 1.$$

*Recalling that* $E\left[e^{g^{1/\beta }}\right]\leq K_{\beta }$, *we have*

(78) $$\int_{1}^{y}g\left(x\right)x^{k}x^{\alpha }\;dx\leq C_{3}y^{k+\beta +\alpha +1},\;\forall y\geq 1.$$

*When* k+β+α+1=0, *we have*

(79) $$\int_{1}^{y}g\left(x\right)x^{k}x^{\alpha }\;dx\leq C_{1}\log y+C_{2}\int_{1}^{y}e^{g{\left(x\right)}^{1/\beta }}x^{\alpha }e^{-x}\;dx,\;\forall y\geq 1.$$

*Again by* $E\left[e^{g^{1/\beta }}\right]\leq K_{\beta }$,

(80) $$\int_{1}^{y}g\left(x\right)x^{k}x^{\alpha }\;dx\leq C_{1}\log y+C_{2},\;\forall y\geq 1.$$

**Proof.**  By using Equation (70), we have

$$\begin{array}{ll} \int_{1}^{y}g\left(x\right)x^{k}x^{\alpha }\;dx & =\int_{1}^{y}\left(e^{x}g\left(x\right)\right)e^{-x}x^{k}x^{\alpha }\;dx\\ & \leq A_{\beta }\int_{1}^{y}\left\{e^{g{\left(x\right)}^{1/\beta }}+e^{x}\log_{+}^{\beta }e^{x}\right\}x^{k}x^{\alpha }e^{-x}\;dx\;\left(∵\mathrm{Equation}\;(70)\right)\\ & \leq A_{\beta }\int_{1}^{y}\left\{x^{k+\beta +\alpha }+e^{g{\left(x\right)}^{1/\beta }}x^{k+\alpha }e^{-x}\right\}\;dx\\ & \leq A_{\beta }\frac{1}{k+\beta +\alpha +1}{\left[x^{k+\beta +\alpha +1}\right]}_{1}^{y}+A_{\beta }\int_{1}^{y}e^{g{\left(x\right)}^{1/\beta }}x^{k+\beta +\alpha +1}x^{-\beta -\alpha -1}x^{\alpha }e^{-x}\;dx\\ & \leq A_{\beta }\frac{1}{k+\beta +\alpha +1}y^{k+\beta +\alpha +1}+A_{\beta }y^{k+\beta +\alpha +1}\int_{1}^{y}e^{g{\left(x\right)}^{1/\beta }}x^{\alpha }e^{-x}\;dx. \end{array}$$

When k+β+α+1=0, in the computation above, we just need to note that the primitive function of $x^{-1}$ is logx. ☐

Of course, when k+β+α+1<0, the left-hand side of Equation (77) is bounded.

We have seen the asymptotic behavior of integrals as y→∞. We can also get the asymptotics as y→0. We will prove this by reducing to the previous case.

**Proposition** **14.** *Suppose that* α>−1, β>0. *Then, there exist constants* $C_{1}$, $C_{2}$ *so that*

(81) $$\int_{0}^{y}g\left(x\right)x^{\alpha }\;dx\leq C_{1}y^{\alpha +1}\int_{0}^{y}e^{g{\left(x\right)}^{1/\beta }}x^{\alpha }\;dx+C_{2}y^{\alpha +1}{\left(-\log y+1\right)}^{\beta },\;\forall y\leq 1.$$

*By using* $E\left[e^{g^{1/\beta }}\right]\leq A_{\beta }$, *we have*

(82) $$\int_{0}^{y}g\left(x\right)x^{\alpha }\;dx\leq Cy^{\alpha +1}{\left(-\log y+1\right)}^{\beta }\;\forall y\leq 1.$$

**Proof.**  For y≤1, we have

$$\begin{array}{ll} \int_{0}^{y}g\left(x\right)x^{\alpha }\;dx & =\int_{0}^{y}\left(g\left(x\right)x^{-\alpha -1}\right)x^{\alpha +1}x^{\alpha }\;dx\\ & \leq A_{\beta }\int_{0}^{y}\left(e^{g{\left(x\right)}^{1/\beta }}+x^{-\alpha -1}\log_{+}^{\beta }x^{-\alpha -1}\right)x^{\alpha +1}x^{\alpha }\;dx\;\left(∵\mathrm{Equation}\;(70)\;\right)\\ & =A_{\beta }\int_{0}^{y}e^{g{\left(x\right)}^{1/\beta }}x^{\alpha +1}x^{\alpha }\;dx+A_{\beta }\int_{0}^{y}x^{-\alpha -1}\left(\alpha +1\right){\left(-\log x\right)}^{\beta }x^{\alpha +1}x^{\alpha }\;dx\\ & \leq A_{\beta }y^{\alpha +1}\int_{0}^{y}e^{g{\left(x\right)}^{1/\beta }}x^{\alpha }\;dx+A_{\beta }\left(\alpha +1\right)\int_{0}^{y}{\left(-\log x\right)}^{\beta }x^{\alpha }\;dx\\ & \leq A_{\beta }y^{\alpha +1}\int_{0}^{y}e^{g{\left(x\right)}^{1/\beta }}x^{\alpha }\;dx+A_{\beta }\left(\alpha +1\right)C{\left(-\log y+1\right)}^{\beta }y^{\alpha +1},\;\left(∵\mathrm{Equation}\;(73)\right) \end{array}$$

which is the desired result. ☐

Lastly, we show the estimate involving the function *f*.

**Proposition** **15.** *We have the following inequality for f*.

(83) $$\int_{1}^{\infty }x^{\beta }f\left(x\right)x^{\alpha }e^{-x}\;dx\leq C_{1}+C_{2}\int_{1}^{\infty }f\left(x\right)\log_{+}^{\beta }f\left(x\right)\;x^{\alpha }e^{-x}\;dx.$$

**Proof.**  From Young’s inequality

$$\begin{array}{ll} \int_{1}^{\infty }x^{\beta }f\left(x\right)x^{\alpha }e^{-x}\;dx & =2^{\beta }\int_{1}^{\infty }{\left(x/2\right)}^{\beta }f\left(x\right)x^{\alpha }e^{-x}\;dx\\ & \leq 2^{\beta }A_{\beta }\int_{1}^{\infty }\left\{e^{{\left({\left(x/2\right)}^{\beta }\right)}^{1/\beta }}+f\left(x\right)\log_{+}^{\beta }f\left(x\right)\right\}x^{\alpha }e^{-x}\;dx\;\left(∵\mathrm{Equation}\;(70)\;\right)\\ & \leq 2^{\beta }A_{\beta }\int_{1}^{\infty }e^{x/2}x^{\alpha }e^{-x}\;dx+2^{\beta }A_{\beta }\int_{1}^{\infty }f\left(x\right)\log_{+}^{\beta }f\left(x\right)x^{\alpha }e^{-x}\;dx\\ & \leq 2^{\beta }A_{\beta }\int_{1}^{\infty }x^{\alpha }e^{-x/2}\;dx+2^{\beta }A_{\beta }\int_{1}^{\infty }f\left(x\right)\log_{+}^{\beta }f\left(x\right)x^{\alpha }e^{-x}\;dx\\ & \leq C_{1}+C_{2}\int_{1}^{\infty }f\left(x\right)\log_{+}^{\beta }f\left(x\right)\;x^{\alpha }e^{-x}\;dx. \end{array}$$

 ☐

We now investigate the spectrum. Let us start with the point spectrum.

**Theorem** **3.** *The point spectrum of* A is {z;ℜz<−β}∪{0,−1,−2,⋯,−[β]}. *Here*, [β] *stands for the integer part of β*.

**Proof.**  We show that *a* is an eigenvalue of A if ℜa<−β. We have seen that M(x)=M(a,α+1;x) satisfies the differential equation AM=aM (see Equation (42)). We only need to show that $M\in L\log^{\beta }L$. The integrability of $\Phi_{\beta }\left(M\right)$ on [0,1] is trivial since *M* is bounded on [0,1]. We see the integrability on [1,∞):

$$\begin{array}{ll} \int_{1}^{\infty }\left|M\left(x\right)\right|\log_{+}^{\beta }\left|M\left(x\right)\right|x^{\alpha }e^{-x}\;dx & ≲\int_{1}^{\infty }e^{x}x^{ℜa-1-\alpha }\left(x+|ℜa-1-\alpha \right|\log_{+}{x)}^{\beta }x^{\alpha }e^{-x}\;dx\\ & ≲\int_{1}^{\infty }x^{ℜa-1}x^{\beta }\;dx\\ & ≲\int_{1}^{\infty }x^{ℜa-1+\beta }\;dx<\infty . \end{array}$$

The finiteness in the last line follows from ℜa−1+β<−1.

It remains to be shown that −n (0≤n<β) is an eigenvalue. In fact, M(−n,α+1;x) is an polynomial of order *n* (a Laguerre polynomial up to normalization) and hence the integrability of $\Phi_{\beta }\left(M\right)$ follows easily.

**Theorem** **4.** *If* ℜa>−β, then a belongs to the resolvent set except when a=0, −1, −2,⋯, −[β].

**Proof.**  We show that $G_{a}$ in (51) is bounded in $L\log^{\beta }L$. To do this, we use Corollary 1. We recall the kernel $G_{a}\left(x,y\right)$.

(1) The case y<x.

Let us consider the resolvent kernel $M\left(y\right)U\left(x\right)y^{\alpha }e^{-y}$ (recall Equation (53) but we ignore a constant multiplication). In the region of x≤1, we have

$$\begin{array}{ll} \int_{0}^{1} & g\left(x\right)x^{\alpha }e^{-x}\;dx\int_{0}^{x}\left|M\left(y\right)U\left(x\right)||f\left(y\right)\right|y^{\alpha }e^{-y}\;dy\\ & ≲\int_{0}^{1}g\left(x\right)x^{\alpha }e^{-x}\;dx\int_{0}^{x}x^{-\alpha }\left|f\left(y\right)\right|y^{\alpha }e^{-y}\;dy\;\left(∵M\;\mathrm{is}\;\mathrm{bounded}\;\mathrm{and}\;\left|U\right|≲x^{-\alpha }\;\mathrm{on}\;\left[0,1\right]\right)\\ & ≲\int_{0}^{1}g\left(x\right)x^{-\alpha }x^{\alpha }e^{-x}\;dx\int_{0}^{1}\left|f\left(y\right)\right|y^{\alpha }e^{-y}\;dy\\ & ≲\int_{0}^{1}\left\{e^{g{\left(x\right)}^{1/\beta }}+x^{-\alpha }\alpha^{\beta }{\left(-\log x\right)}^{\beta }\right\}x^{\alpha }e^{-x}\;dx\int_{0}^{1}\left|f\left(y\right)\right|\log_{+}^{\beta }\left|f\left(y\right)\right|y^{\alpha }e^{-y}\;dy\\ & \;\;(∵\;x≲x\log_{+}^{\beta }x\;\mathrm{and}\;\mathrm{Equation}\;(70))\\ & ≲\int_{0}^{1}\left|f\left(y\right)\right|\log_{+}^{\beta }\left|f\left(y\right)\right|y^{\alpha }e^{-y}\;dy. \end{array}$$

When α=0, |U|≲−logx in the above computation should be changed as follows:

$$\begin{array}{ll} \int_{0}^{1} & g\left(x\right)e^{-x}\;dx\int_{0}^{x}\left|M\left(y\right)U\left(x\right)||f\left(y\right)\right|e^{-y}\;dy\\ & ≲\int_{0}^{1}g\left(x\right)e^{-x}\;dx\int_{0}^{x}\left|\log x||f\left(y\right)\right|e^{-y}\;dy\\ & ≲\int_{0}^{1}g\left(x\right)|\log x|e^{-x}\;dx\int_{0}^{1}\left|f\left(y\right)\right|e^{-y}\;dy\\ & ≲\int_{0}^{1}\{e^{g{\left(x\right)}^{1/\beta }}+\left|\log x\right|\log_{+}^{\beta }\left|\log x|\right\}e^{-x}\;dx\int_{0}^{1}\left|f\left(y\right)\right|\log_{+}^{\beta }\left|f\left(y\right)\right|e^{-y}\;dy\\ & \;(∵\;x≲x\log_{+}^{\beta }x\;\mathrm{and}\;\mathrm{Equation}\;(70))\\ & ≲\int_{0}^{1}\left|f\left(y\right)\right|\log_{+}^{\beta }\left|f\left(y\right)\right|e^{-y}\;dy. \end{array}$$

In the region of x≥1, we have

$$\begin{array}{ll} \int_{1}^{\infty } & g\left(x\right)x^{\alpha }e^{-x}\;dx\int_{0}^{x}\left|M\left(y\right)U\left(x\right)||f\left(y\right)\right|y^{\alpha }e^{-y}\;dy\\ & ≲\int_{1}^{\infty }x^{-ℜa}g\left(x\right)x^{\alpha }e^{-x}\;dx\int_{0}^{x}\left|M\left(y\right)|f\left(y\right)\right|y^{\alpha }e^{-y}\;dy\;\left(∵\;\left|U\right|≲x^{-ℜa}\;\mathrm{on}\;\left[1,\infty )\right]\right)\\ & =\int_{1}^{\infty }x^{-ℜa}g\left(x\right)x^{\alpha }e^{-x}\;dx\int_{0}^{1}\left|M\left(y\right)||f\left(y\right)\right|y^{\alpha }e^{-y}\;dy\\ & \;+\int_{1}^{\infty }x^{-ℜa}g\left(x\right)x^{\alpha }e^{-x}\;dx\int_{1}^{x}\left|M\left(y\right)||f\left(y\right)\right|y^{\alpha }e^{-y}\;dy\\ & ≲\int_{1}^{\infty }x^{-ℜa}g\left(x\right)x^{\alpha }e^{-x}\;dx\int_{0}^{1}\left|f\left(y\right)\right|y^{\alpha }e^{-y}\;dy\\ & \;+\int_{1}^{\infty }x^{-ℜa}g\left(x\right)x^{\alpha }e^{-x}\;dx\int_{1}^{x}e^{y}y^{ℜa-1-\alpha }\left|f\left(y\right)\right|y^{\alpha }e^{-y}\;dy\\ & \;\;(∵\;\left|M\right|\;\mathrm{is}\;\mathrm{bounded}\;\mathrm{on}\;\left[0,1\right]\;\mathrm{and}\;\left|M\right|≲e^{y}y^{ℜa-1-\alpha }\;\mathrm{on}\;\left[1,\infty \right))\\ & ≲\int_{1}^{\infty }x^{-ℜa}g\left(x\right)x^{\alpha }e^{-x}\;dx\int_{0}^{1}\left|f\left(y\right)\right|y^{\alpha }e^{-y}\;dy\\ & \;+\int_{1}^{\infty }y^{ℜa-1}\left|f\left(y\right)\right|\;dy\int_{y}^{\infty }x^{-ℜa}g\left(x\right)x^{\alpha }e^{-x}\;dx\;\left(∵\;\mathrm{Fubini}\text{’}s\;\mathrm{theorem}\right)\\ & ≲\int_{0}^{1}\left|f\left(y\right)\right|y^{\alpha }e^{-y}\;dy+\int_{1}^{\infty }y^{ℜa-1}\left|f\left(y\right)\right|y^{-ℜa}y^{(\alpha +\beta )\vee 0}e^{-y}\;dy\;\left(∵\mathrm{Equation}\;(75)\right)\\ & ≲\int_{0}^{1}\left|f\left(y\right)\right|y^{\alpha }e^{-y}\;dy+\int_{1}^{\infty }\left|f\left(y\right)\right|y^{-1}y^{(\alpha +\beta )\vee 0}e^{-y}\;dy\\ & ≲\int_{0}^{1}\left|f\left(y\right)\right|y^{\alpha }e^{-y}\;dy+\int_{1}^{\infty }\left|f\left(y\right)\right|y^{\alpha +\beta }e^{-y}\;dy\;\left(∵\left(\alpha +\beta \right)\vee 0<\alpha +\beta +1\right)\\ & ≲\int_{0}^{1}\left|f\left(y\right)\right|\log_{+}^{\beta }\left|f\left(y\right)\right|y^{\alpha }e^{-y}\;dy+\int_{1}^{\infty }\left|f\left(y\right)\right|\log_{+}^{\beta }\left|f\left(y\right)\right|y^{\alpha }e^{-y}\;dy.\\ & \;\;(∵\;x≲x\log_{+}^{\beta }x\;\mathrm{and}\;\mathrm{Equation}\;(83)) \end{array}$$

(2) The case y>x.

We consider the resolvent kernel $M\left(x\right)U\left(y\right)y^{\alpha }e^{-y}$. In the region x≤1, we have

$$\begin{array}{ll} \int_{0}^{1} & g\left(x\right)x^{\alpha }e^{-x}\;dx\int_{x}^{\infty }\left|M\left(x\right)U\left(y\right)||f\left(y\right)\right|y^{\alpha }e^{-y}\;dy\\ & ≲\int_{0}^{1}g\left(x\right)x^{\alpha }e^{-x}\;dx\int_{x}^{1}\left|U\left(y\right)||f\left(y\right)\right|y^{\alpha }e^{-y}\;dy\\ & \;+\int_{0}^{1}g\left(x\right)x^{\alpha }e^{-x}\;dx\int_{1}^{\infty }\left|U\left(y\right)||f\left(y\right)\right|y^{\alpha }e^{-y}\;dy\\ & ≲\int_{0}^{1}g\left(x\right)x^{\alpha }e^{-x}\;dx\int_{x}^{1}y^{-\alpha }\left|f\left(y\right)\right|y^{\alpha }e^{-y}\;dy+\int_{0}^{1}g\left(x\right)x^{\alpha }e^{-x}\;dx\int_{1}^{\infty }y^{-ℜa}\left|f\left(y\right)\right|y^{\alpha }e^{-y}\;dy\\ & \;\;(∵\;\left|U\right|≲y^{-\alpha }\;\mathrm{on}\;\left[0,1\right],\;\left|U\right|≲y^{-ℜa}\;\mathrm{on}\;\left[1,\infty )\right])\\ & ≲\int_{0}^{1}\left|f\left(y\right)\right|e^{-y}\;dy\int_{0}^{y}g\left(x\right)x^{\alpha }e^{-x}\;dx+\int_{1}^{\infty }y^{-ℜa}\left|f\left(y\right)\right|y^{\alpha }e^{-y}\;dy\\ & \;\;(∵\;\mathrm{Fubini}\text{’}s\;\mathrm{theorem}\;\mathrm{and}\;\mathrm{Equation}\;(82))\;\mathrm{for}\;y=1)\\ & ≲\int_{0}^{1}\left|f\left(y\right)\right|e^{-y}y^{\alpha +1}{\left(-\log y+1\right)}^{\beta }\;dy+\int_{1}^{\infty }y^{\beta }\left|f\left(y\right)\right|y^{\alpha }e^{-y}\;dy\;\left(∵\mathrm{Equation}\;(82))\;\mathrm{and}\;ℜa>-\beta \right)\\ & ≲\int_{0}^{1}\left|f\left(y\right)\right|y^{\alpha }e^{-y}\;dy+\int_{1}^{\infty }\left|f\left(y\right)\right|\log_{+}^{\beta }\left|f\left(y\right)\right|y^{\alpha }e^{-y}\;dy\\ & \;\;(∵\;y{\left(-\log y+1\right)}^{\beta }\;\mathrm{is}\;\mathrm{bounded}\;\mathrm{on}\;\left[0,1\right]\;\mathrm{and}\;\mathrm{Equation}\;(83))\\ & ≲\int_{0}^{1}\left|f\left(y\right)\right|\log_{+}^{\beta }\left|f\left(y\right)\right|y^{\alpha }e^{-y}\;dy+\int_{1}^{\infty }\left|f\left(y\right)\right|\log_{+}^{\beta }\left|f\left(y\right)\right|y^{\alpha }e^{-y}\;dy\;\left(∵\;x≲x\log_{+}^{\beta }x\;\right). \end{array}$$

In the region x≥1, we have

$$\begin{array}{ll} \int_{1}^{\infty }g\left(x\right)x^{\alpha }e^{-x}\;dx & \int_{x}^{\infty }\left|M\left(x\right)U\left(y\right)||f\left(y\right)\right|y^{\alpha }e^{-y}\;dy\\ & ≲\int_{1}^{\infty }g\left(x\right)x^{\alpha }e^{-x}\;dx\int_{x}^{\infty }e^{x}x^{ℜa-1-\alpha }y^{-ℜa}\left|f\left(y\right)\right|y^{\alpha }e^{-y}\;dy\\ & \;\;(∵\;\left|M\right|≲e^{x}x^{ℜa-1-\alpha }\;\mathrm{and}\;\left|U\right|≲y^{-ℜa}\;\mathrm{on}\;\left[1,\infty \right))\\ & ≲\int_{1}^{\infty }y^{-ℜa}y^{\alpha }\left|f\left(y\right)\right|e^{-y}\;dy\int_{1}^{y}g\left(x\right)x^{ℜa-1}\;dx\;\left(∵\;\mathrm{Fubini}\text{’}s\;\mathrm{theorem}\right)\\ & ≲\int_{1}^{\infty }y^{-ℜa}y^{\alpha }\left|f\left(y\right)\right|e^{-y}y^{ℜa-1+\beta +1}\;dy\;\left(∵\mathrm{Equation}\;(78)\right)\\ & ≲\int_{1}^{\infty }y^{\beta }\left|f\left(y\right)\right|y^{\alpha }e^{-y}\;dy\\ & ≲\int_{1}^{\infty }\left|f\left(y\right)\right|\log_{+}^{\beta }\left|f\left(y\right)\right|y^{\alpha }e^{-y}\;dy\;\left(∵\mathrm{Equation}\;(83)\right) \end{array}$$

Thus, we have shown that $G_{a}$ is bounded in $L\log^{\beta }L$. Hence, the spectrum of A is completely determined. ☐

**Figure 1:**
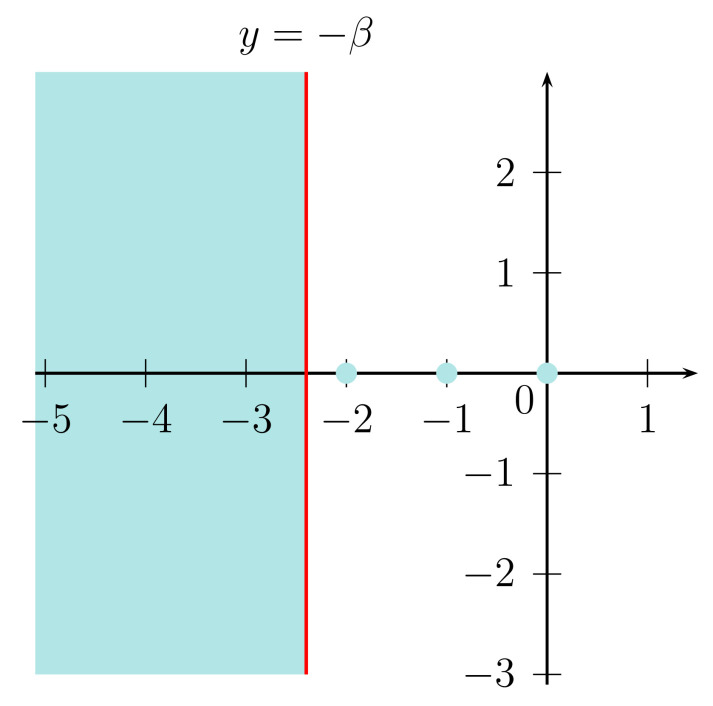
The spectrum in the space $L\log^{\beta }L$.

The spectrum is shown as in Figure 1. The case of β=1 is the Zygmund space. Hence, the spectral gap equals 1. So we have $\zeta_{\mathrm{SG}}=1$ where $\zeta_{\mathrm{SG}}$ is defined by Equation (33). Therefore, by Equation (34), we have

$$\begin{array}{c} \frac{1}{2}=\gamma_{\mathrm{LS}}\leq \gamma_{Z\to Z}\leq \zeta_{\mathrm{SG}}=1 \end{array}$$

and so it shows that $\gamma_{\mathrm{LS}}\neq \zeta_{\mathrm{SG}}$ may happen. This is a well-known result in the case of $L^{2}$. Furthermore, in [9], we have shown that assuming the logarithmic Sobolev inequality, the spectra in $L^{p}$ (1<p<∞) are all the same. In our case, the spectrum in $L^{2}$ is 0, −1, −2,⋯ The spectrum in $L\log^{\beta }L$ is quite different from that. Moreover, the logarithmic Sobolev inequality may not give a uniform estimate for spectral gaps among the Orlicz spaces $L\log^{\beta }L$ (β>0).
